# Supplementary material for: NKX2-5 regulates human cardiomyogenesis via a HEY2 dependent transcriptional network
Source: Nat Commun. 2018 Apr 10;9:1373. doi: 10.1038/s41467-018-03714-x (PMC5893543; doi:10.1038/s41467-018-03714-x)
Supplement: Supplementary file 1 — Supplementary Information(PDF 9342 kb) [file 41467_2018_3714_MOESM1_ESM.pdf]

Supplementary Information

NKX2-5 regulates human cardiomyogenesis via a *HEY2*-dependent transcriptional network

Anderson et al.

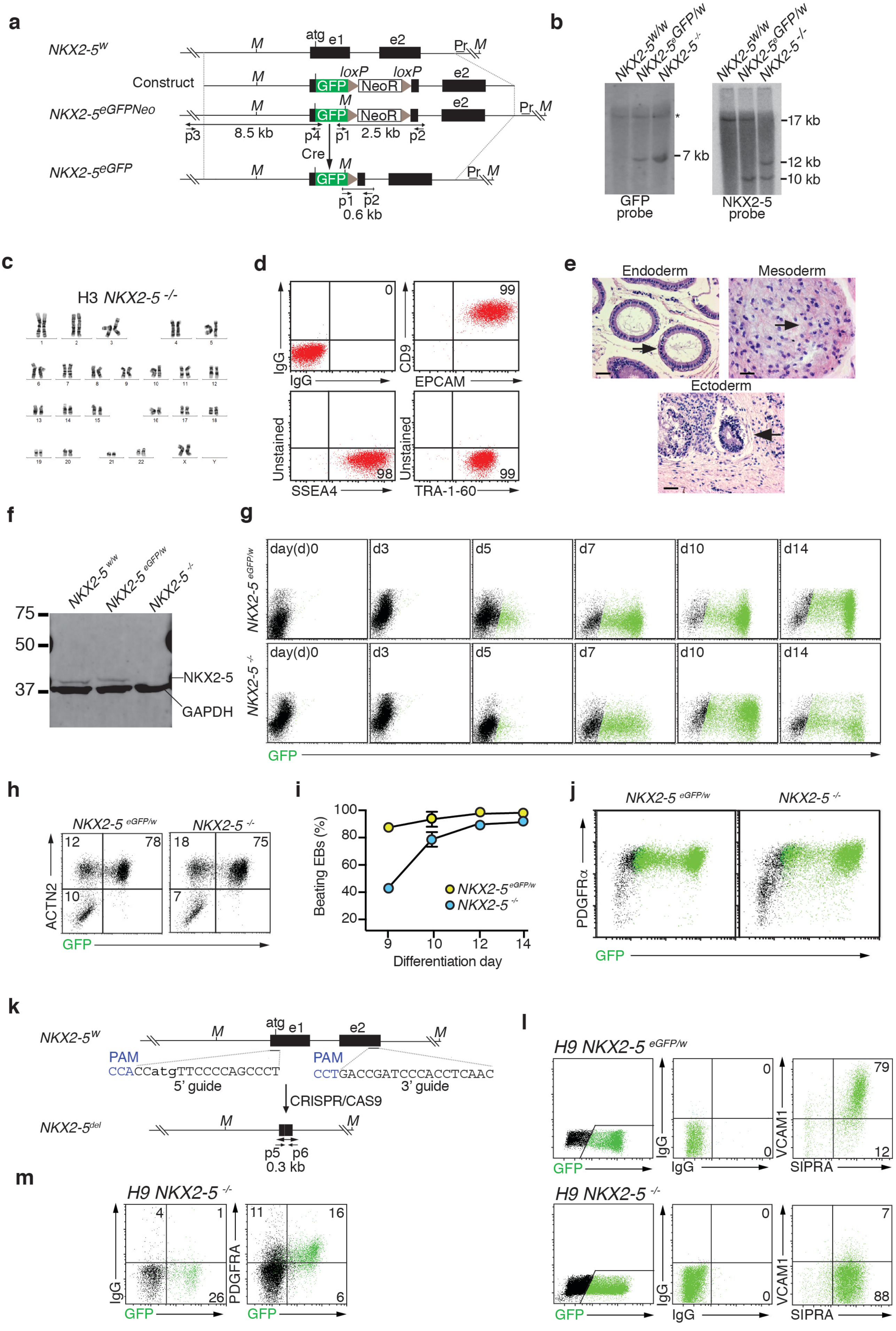
a
NKX2-5w
atg e1
e2
M
Pr
Construct
loxP
GFP
NeoR
NKX2-5eGFPNeo
p3
8.5 kb
p4
p1
2.5 kb
p2
Cre
NKX2-5eGFP
0.6 kb
b
NKX2-5w/w
NKX2-5eGFP/w
NKX2-5-/-
7 kb
17 kb
12 kb
10 kb
GFP probe
NKX2-5 probe
c
H3 NKX2-5 -/-
d
IgG
CD9
EPCAM
Unstained
SSEA4
TRA-1-60
0
99
98
99
e
Endoderm
Mesoderm
Ectoderm
f
75
50
37
NKX2-5
GAPDH
g
NKX2-5 eGFP/w
NKX2-5 -/-
day(d)0
d3
d5
d7
d10
d14
GFP
h
ACTN2
12
78
10
18
75
7
i
Beating EBs (%)
Differentiation day
j
PDGFRα
k
PAM
CCACCatgTTCCCCAGCCCT
5' guide
CCTGACCGATCCCACCTCAAC
3' guide
CRISPR/CAS9
NKX2-5del
p5
p6
0.3 kb
l
H9 NKX2-5 eGFP/w
H9 NKX2-5 -/-
VCAM1
SIPRA
79
12
7
88
m
PDGFRA
4
1
26
11
16
6

Supplementary Figure 1. Generation and characterisation of *NKX2-5* null human embryonic stem cells.

(**a**) Schematic of targeting strategy to target second *NKX2-5* allele in *NKX2-5*$^{eGFP/w}$ hESCs. Boxes denote exons. Positions of the *MluI* (M) restriction sites, the 3' probe (Pr) and the primers (p1-p4) used to map the structure of the modified locus are shown. eGFP - enhanced Green Fluorescent Protein.

(**b**) Southern blot showing correct integration of eGFP (left blot) and locus organisation (right blot) at NKX2-5 locus. * non-specific band.

(**c**) Karyogram from NKX2-5$^{-/-}$ hESCs shows normal chromosomal complement.

(**d**) Flow cytometry demonstrating that *NKX2-5*$^{-/-}$ hESCs retain expression of pluripotency markers TRA-1-60, CD9, EPCAM and SSEA4. Numbers on each plot show the percentage of cells found in the quadrants indicated. These data demonstrate that 98 % of NKX2-5 null cardiomyocytes express these markers.

(**e**) Hematoxylin and eosin stained histological sections of teratomas from *NKX2-5*$^{-/-}$ hESCs showing derivatives of ectoderm, mesoderm and endoderm. Arrows denote structures that derive from the three germ layers; neural rosettes (ectoderm), cartilage (mesoderm) and secretory columnar epithelium (endoderm).

(**f**) Western blot to detect NKX2-5 from wildtype (*NKX2-5*$^{w/w}$), *NKX2-5*$^{eGFP/+}$ and *NKX2-5*$^{-/-}$ cultures at day 14 of differentiation demonstrates that *NKX2-5* heterozygote and wildtype *NKX2-5* cardiomyocytes have comparable NKX2-5 levels and *NKX2-5* null cardiomyocytes do not express NKX2-5.

(**g**) Flow cytometry demonstrates similar kinetics of GFP expression during cardiac differentiation of both *NKX2-5*$^{eGFP/+}$ and *NKX2-5*$^{-/-}$ hESCs.

(**h**) Flow cytometry shows that all GFP^{+} cells express ACTN2 (cardiac α-actinin) in *NKX2-5*$^{eGFP/+}$ and *NKX2-5*$^{-/-}$ cultures at day 14 of differentiation. Numbers on each plot show the percentage of cells found in the quadrants indicated.

(**i**) Quantification of contractility during embryoid body differentiation of *NKX2-5*$^{eGFP/w}$ and *NKX2-5*$^{-/-}$ hESCs demonstrates *NKX2-5* null cultures have impaired cardiomyogenesis, as demonstrated by a delayed onset of rhythmic contraction. Mean ± SEM (n = 4).

(**j**) Flow cytometry demonstrates PDGFRα expression at day 14 of monolayer differentiation is equivalent in *NKX2-5*$^{eGFP/w}$ and *NKX2-5*$^{-/-}$ cultures.

(**k**) Schematic of CRISPR/Cas9 mediated mutation of *NKX2-5* locus in the H9 cell line. eGFP was introduced into one *NKX2*-5 allele using the targeting strategy outlined in **a.** The coding sequence of the second *NKX2-5* allele was deleted resulting from Non-Homologous End Joining (NHEJ) after CRISPR/Cas9 treatment.

(**l**) Flow cytometric analysis of H9 *NKX2-5*$^{-/-}$ (i.e. *NKX2-5*$^{eGFP/del}$) cultures at day 14 of differentiation. VCAM1 expression is greatly reduced in H9 NKX2-5 deficient cardiomyocytes, consistent with the HES3 phenotype. SIRPA expression is unperturbed in the *NKX2-5* knockout. Numbers on plots show the percentage of cells found in the quadrants indicated.

(**m**) Flow cytometry demonstrates that PDGFRA expression is maintained in H9 *NKX2-5*$^{-/-}$ cultures at day 35 of culture. This phenotype is consistent with data obtained from the HES3 cell line. Numbers on plots show the percentage of cells found in the quadrants indicated.

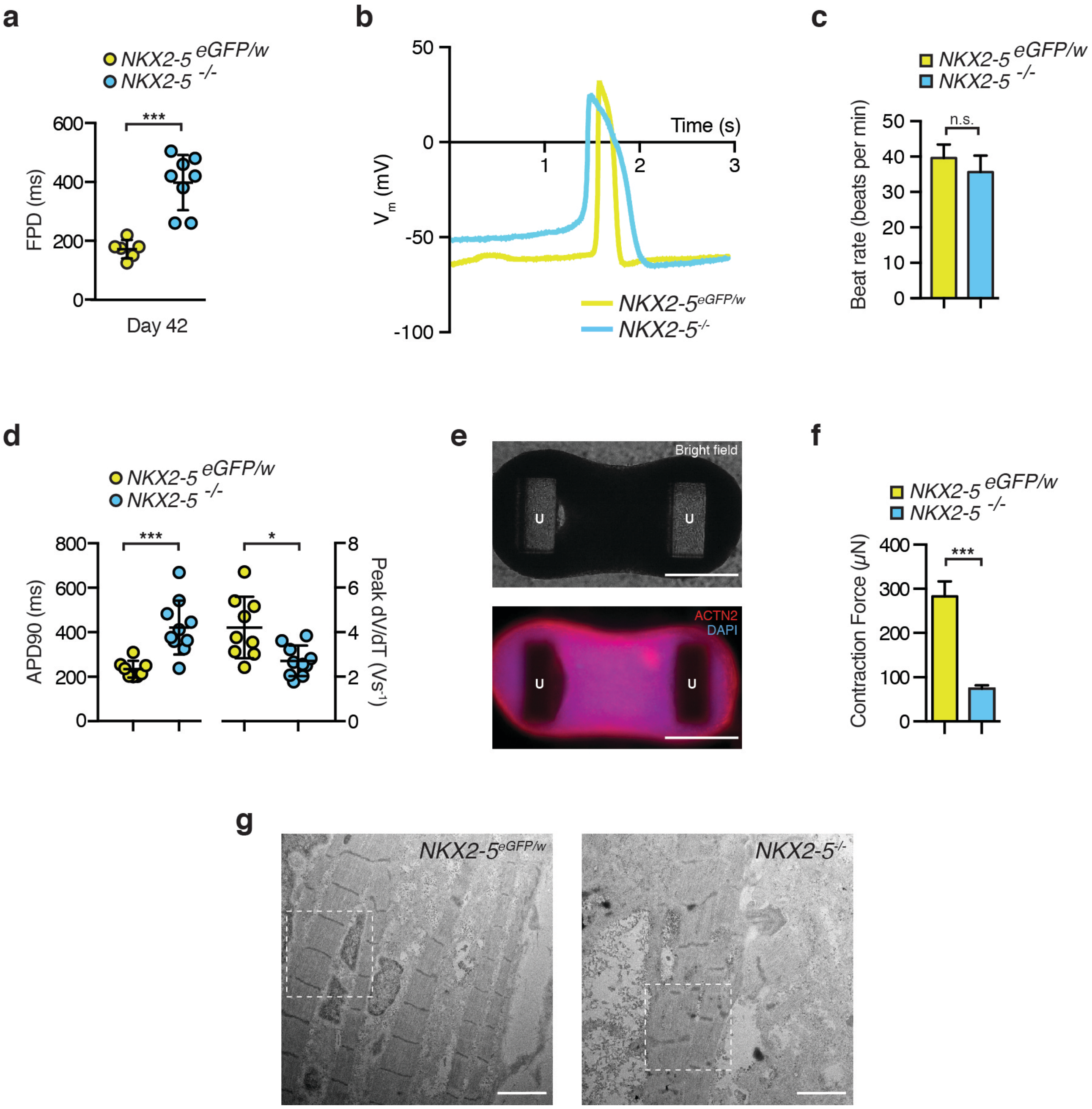

Supplementary Figure 2. Functional analysis of *NKX2-5*$^{-/-}$ cardiomyocytes.

(**a**) Dot plots of field potential duration (FPD) of cardiomyocyte aggregates derived from *NKX2-5*$^{eGFP/w}$ and *NKX2-5*$^{-/-}$ cultures at day 42 of differentiation. Bars represent mean ± SD (n = 8). *** p<0.001 (Student's t-test).

(**b**) Representative whole-cell patch clamp traces of action potentials derived from single *NKX2-5*$^{eGFP/w}$ and *NKX2-5*$^{-/-}$ cardiomyocytes at day 14 of differentiation.

(**c**) Bar graph demonstrating individual *NKX2-5*$^{eGFP/w}$ and *NKX2-5*$^{-/-}$ cardiomyocytes have similar rates of contraction at day 14 of differentiation, as determined by whole-cell patch clamp. Mean ± SEM (n = 8).

(**d**) Dot plots of action potential duration (APD90) and upstroke velocity (dV/dT) derived from whole-cell patch clamp. *NKX2-5* null cardiomyocytes have a reduced upstroke velocity and prolonged APD90. Bars represent mean ± SD (n = 8). * p<0.05, *** p<0.001 (Student's t-test).

(**e**) Bright field and epifluorescence images of bioengineered cardiac organoids used to assess contractile force. Epifluorescent image captures cardiac α-actinin (ACTN2) and nuclear DAPI staining. U = polymer uprights around which cardiac organoids form. Scale bar = 500 μM

(**f**) Bar graph showing contractile force produced by *NKX2-5*$^{eGFP/w}$ and *NKX2-5*$^{-/-}$ bioengineered cardiac organoids. Data represent mean ± SEM (n = 12). *** p<0.001 (Student's t-test).

(**g**) Transmission electron micrographs show that *NKX2-5* null cardiomyocytes have disorganised sarcomeres compared to *NKX2-5*$^{eGFP/w}$ cardiomyocytes. Dashed boxes are shown figure 2. Scale bar = 1 μM.

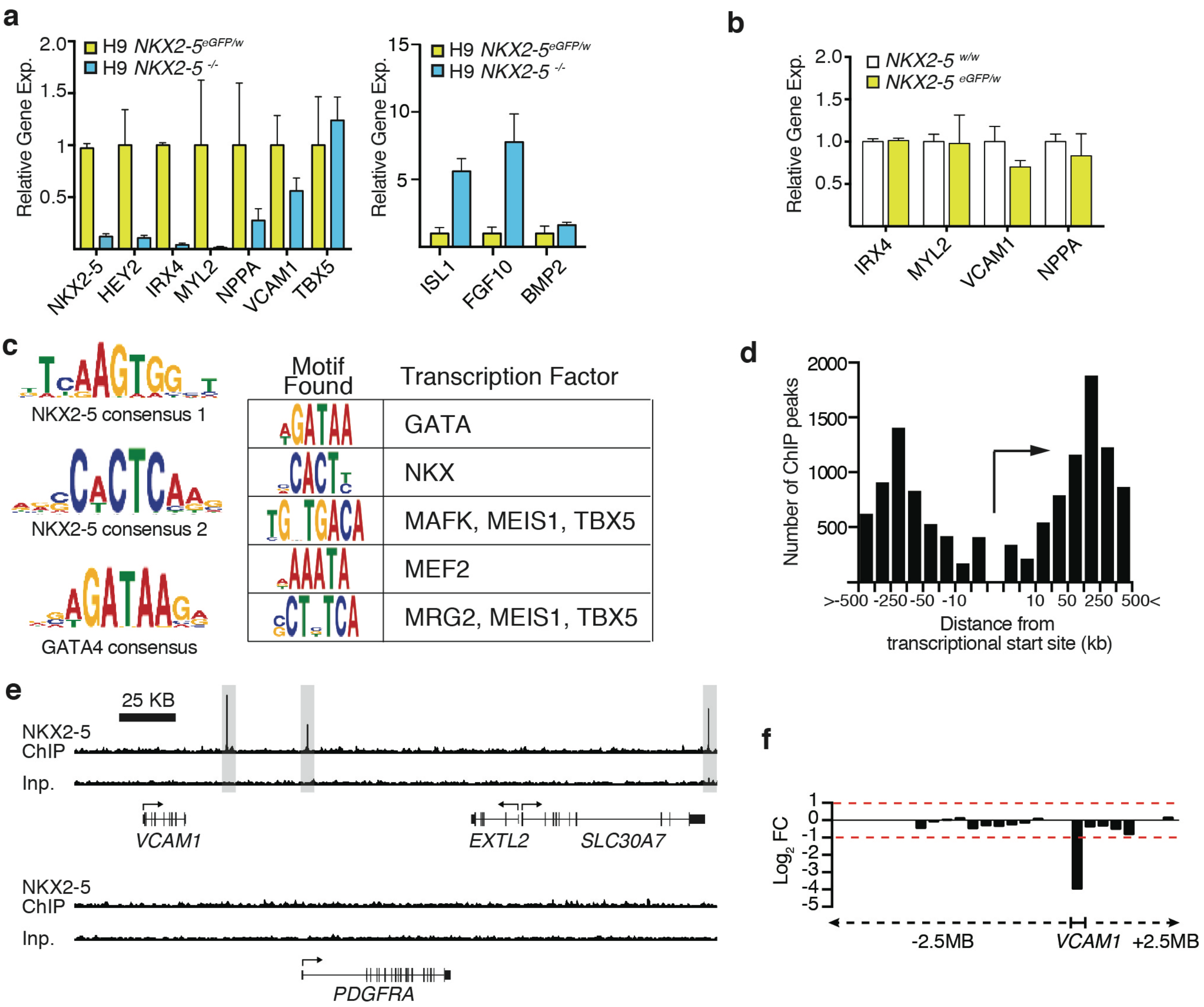

Supplementary Figure 3. NKX2-5 directly regulates key cardiomyogenic markers.

(**a**) Q-PCR analysis of H9 *NKX2-5*$^{eGFP/w}$ and *NKX2-5*$^{-/-}$ cultures at day 14 of differentiation. H9 *NKX2-5* null cardiomyocytes show reduced transcript levels for *NKX2-5*, *HEY2, IRX4*, *MYL2*, *NPPA* and increased *ISL1*, *FGF10* and *BMP2*. Levels of TBX5 are consistent between H9 *NKX2-5* heterozygotes and null cardiomyocytes. These data match the gene expression profile observed in HES3 cells (Figure 3). Data are presented as gene expression relative to H9 *NKX2-5*$^{eGFP/w}$ and represent mean ± SEM (n = 3-6).

(**b**) Bar graph of Q-PCR analysis *NKX2-5*$^{eGFP/w}$ and wildtype cardiomyocytes. Data are presented as gene expression relative to wildtype (*NKX2-5*$^{w/w}$) and represent mean ± SEM (n = 3).

(**c**) Transcription factor binding motifs identified within NKX2-5 ChIP-seq bound regions determined by MEME CentriMo analysis. These included two NKX2-5 consensus motifs, GATA motifs and TBX5 recognition sites.

(**d**) Distribution of NKX2-5 bound regions relative to transcriptional start sites (TSS). The data shows bimodal distribution with most sites located between 50 to 500 kb from TSS suggesting NKX2-5 acts at distant enhancers.

(**e**) Representative schematic of NKX2-5 ChIP-seq tracks at *VCAM1* and *PDGFRA* loci show that NKX2-5 is bound at the *VCAM1* locus. NKX2-5 binding site shaded grey. Inp = Input chromatin.

(**f**) Bar graph showing differential expression of all genes 2.5 Mbp up or downstream of the *VCAM1* locus in (**c**), as determined from RNA-seq data. This data shows *VCAM1* is the only differentially expressed gene in this chromosomal region. Red dashed line marks 2 fold gene expression difference between genotypes.

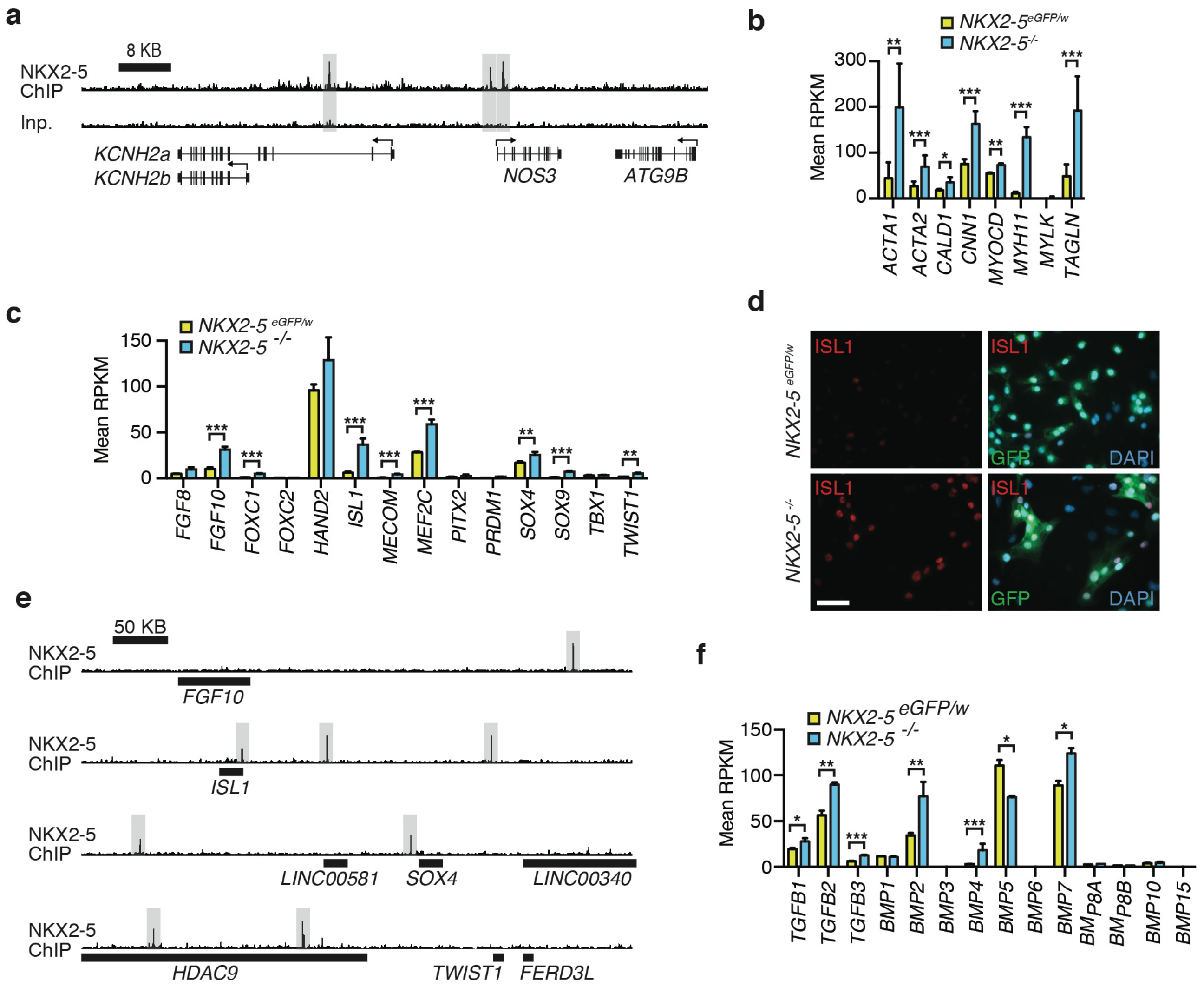

Supplementary Figure 4. Regulation of cardiomyocyte markers and cardiac progenitor genes by NKX2-5

(**a**) Representative schematic of NKX2-5 ChIP-seq tracks at *KCNH2* showing NKX2-5 binding at the *KCNH2* and *NOS3* loci. NKX2-5 site is located in intron 2 of the *KCNH2a* transcript, which may directly activate *KCNH2b.* NKX2-5 binding site shaded grey. Inp. = Input chromatin.

(**b**) Bar graph showing expression of a subset of smooth muscle markers in *NKX2-5*$^{eGFP/w}$ and *NKX2-5*$^{-/-}$ cardiomyocytes as determined from RNA-seq data. RPKM = reads per kilobase million. * p<0.05, ** p<0.01, *** p<0.001 adjusted p-values (Student's t-test).

(**c**) Bar graph showing expression of cardiac progenitor markers and non-cardiomyocyte cardiovascular lineage markers in *NKX2-5*$^{eGFP/w}$ and *NKX2-5*$^{-/-}$ cardiomyocytes as determined by RNA-seq data. Cardiac progenitor markers *ISL1, MEF2C* and *FGF10* are dysregulated as are the atrioventricular canal genes *SOX4* and *TWIST1.* RPKM = reads per kilobase million. ** p<0.01, *** p<0.001 adjusted p-values (Moderated t-test).

(**d**) Immunofluorescent detection of ISL1, GFP and merged images (DAPI stain highlights nuclei) from both *NKX2-5*$^{eGFP/w}$ and *NKX2-5*$^{-/-}$ cardiomyocytes at day 14 of differentiation. Most *NKX2-5*$^{-/-}$ cardiomyocytes maintain ISL1 expression. Scalebar = 50 µm.

(**e**) Representative schematic of NKX2-5 ChIP-seq tracks show NKX2-5 binding sites at the *FGF10*, *ISL1*, *SOX4* and *TWIST1* loci. NKX2-5 binding sites shaded grey.

(**f**) Bar graph showing expression of transforming growth factor β superfamily members in *NKX2-5*$^{eGFP/w}$ and *NKX2-5*$^{-/-}$ cardiomyocytes as determined from RNA-seq data.. These data show that NKX2-5 is required for down regulation of both *BMP2* and *BMP4 in vitro.* RPKM = reads per kilobase million. * p<0.05, ** p<0.01, *** p<0.001 adjusted p-values (Moderated t-test)

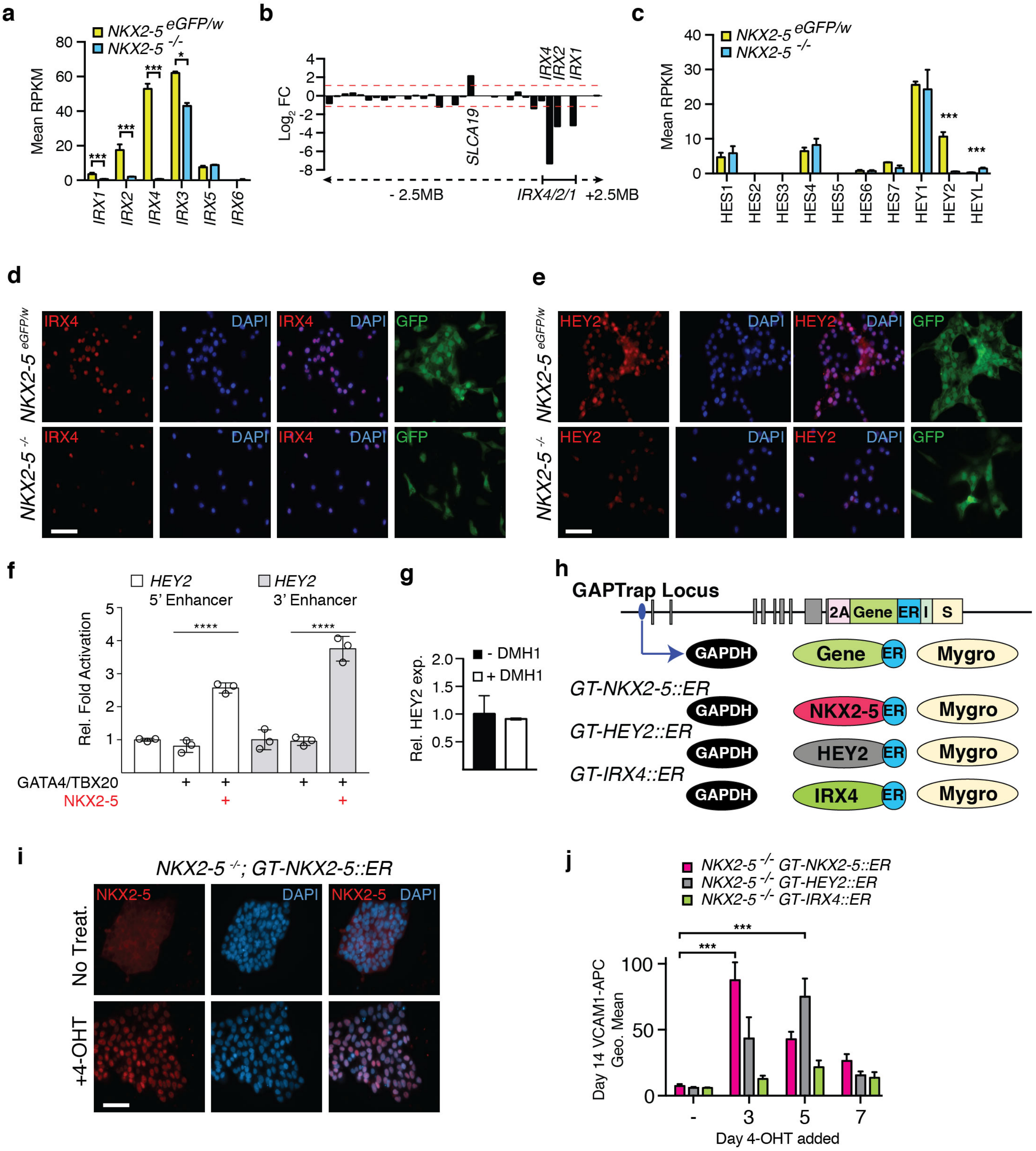

Supplementary Figure 5. Genetic dissection of the NKX2-5-dependant transcriptional network driving human cardiomyogenesis.

(**a**) Bar graph showing expression of all members of the *Iroquois* gene family in *NKX2-5*$^{eGFP/w}$ and *NKX2-5*$^{-/-}$ cardiomyocytes as determined from RNA-seq data. The *IRX1/2/4* cluster is differentially regulated. RPKM = reads per kilobase million. * p<0.05, ** p<0.01, *** p<0.001 adjusted p-values (Moderated t-test).

(**b**) Bar graph showing differential expression of all genes 2.5 Mbp up or downstream of the *IRX1/2/4* locus in Fig. 4C, as determined from RNA-seq data. Red dashed line marks 2 fold gene expression difference between genotypes.

Differential expression of genes 2.5 Mbp up or downstream of the *IRX1/2/4* locus, as determined from RNA-seq data. Red dashed line marks 2 fold (adj. p value <0.05) gene expression difference between genotypes. Log FC = $\log_2$ fold change.

(**c**) Bar graph showing expression of all members of the HES/HEY family of transcription factors in *NKX2-5*$^{eGFP/w}$ and *NKX2-5*$^{-/-}$ cardiomyocytes as determined from RNA-seq data. Only *HEY2* is differentially regulated. RPKM = reads per kilobase million. * p<0.05, ** p<0.01, *** p<0.001 adjusted p-values (Moderated t-test).
(**d** and **e**) Immunofluorescent detection of IRX4 (D), HEY2 (E), DAPI staining of nuclei and GFP in *NKX2-5*$^{eGFP/w}$ and *NKX2-5*$^{-/-}$ cardiomyocytes. Consistent with reduced RNA levels, both IRX4 and HEY2 protein levels are reduced in NKX2-5 null cardiomyocytes. Scalebar = 50 µm.
(f) *In vitro* transcriptional analysis using FLAG-tagged Nkx2-5 expression vectors and a luciferase reporter containing the 5' or 3' HEY2 enhancer elements. The cardiac transcription factors Gata4 and Tbx20a were also included where indicated. Data represent the mean and SEM (n=3) and values of each biological replicate are indicated by circles. **** p<0.0001 (ANOVA).
(**g**) Bar graph of expression of *HEY2* in cardiomyocytes after BMP signaling inhibition using DMH1. *HEY2* levels are not reduced in presence of DMH1.
(**h**) Diagrammatic representation of the modified *GAPDH* locus expressing NKX2-5::ER, HEY2::ER and IRX4::ER fusion proteins. Proteins produced from the modified locus are depicted as ovals. GT = GAPTrap; 2A = T2A cleavage peptide; ER = Estrogen Receptor domain; I = Internal Ribosome Entry Site (IRES); S = selectable marker, Mygro = Hygromyocin Resistance gene designated Mygro as codon usage has been optimized for mammalian cells.
(**i**) Immunofluorescent detection of the NKX2-5::ER fusion in GT-NKX2-5::ER hESCs using an antibody to NKX2-5, nuclei are counterstained with DAPI. Upon addition of the estrogen analogue, 4OHT, the NKX2-5::ER fusion protein relocates to the nucleus (lower panels). Scalebar = 50 µm.
(**j**) Bar graphs showing quantification of flow cytometric analysis of day 14 VCAM1 expression (see Fig. 4F). The day of 4 OHT addition is indicated on the X-axis. Geo. Mean = geometric mean fluorescence. Data represent mean ± SEM (n = 4). *** p<0.001 (Student's t-test).

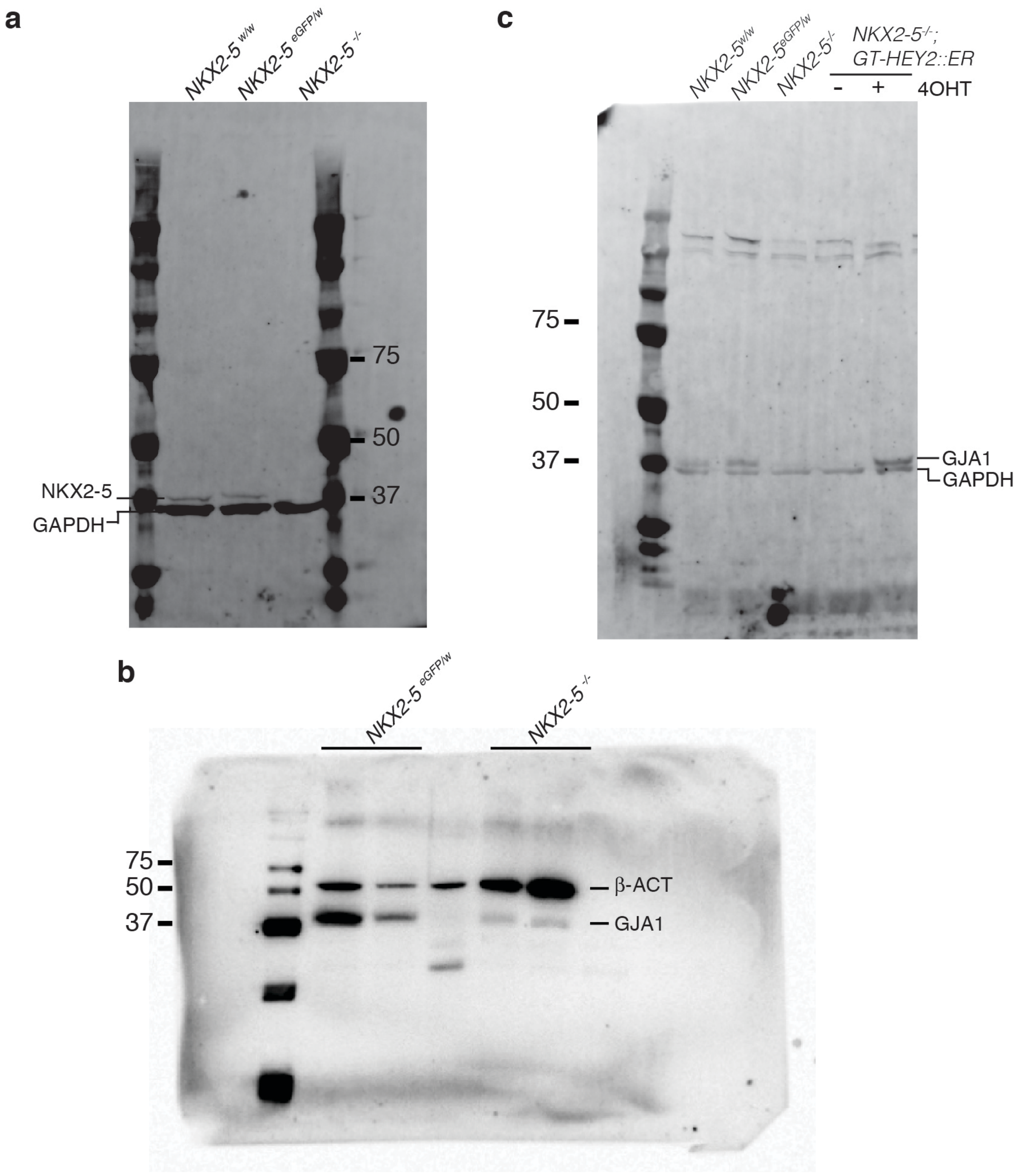

Supplementary Figure 6. Uncropped Western Blots of NKX2-5, GJA1, GAPDH and β-actin. (**a**) Western blot shown in Supplementary Fig. 1 f to detect NKX2-5 from wildtype (*NKX2-5$^{w/w}$*), *NKX2-5$^{eGFP/+}$* and *NKX2-5$^{-/-}$* cardiomyocytes at day 14. GAPDH is used as a loading control. Bands denoting NKX2-5 and GAPDH are indicated to left of blot. Molecular weight markers for 37, 50 and 75 kDa are indicated to the left.
(**b**) Western blot shown in Fig. 4d to detect GJA1 from *NKX2-5$^{eGFP/+}$* and *NKX2-5$^{-/-}$* cardiomyocytes at differentiation day 14. β-actin is used as a loading control. Bands denoting NKX2-5 and β-actin are indicated to the right of blot. Molecular weight markers for 37, 50 and 75 kDa are indicated to the left. M = lane containing molecular weight markers.
(**c**) Western blot shown in Fig. 5i to detect of GJA1 (connexin 43) levels in wildtype (*NKX2-5$^{w/w}$*), *NKX2-5$^{eGFP/+}$*, *NKX2-5$^{-/-}$* and *NKX2-5$^{-/-}$;GT-NKX2-5::ER* (- or + 4OHT) day 14 cardiomyocytes.

GAPDH is used as a loading control. GJA1 and GAPDH bands are indicated to the right of the panel. Molecular weight makers for 37, 50 and 75 kDa are indicated to the left. M = lane containing molecular weight markers.

Supplementary Methods

Whole-Cell Patch Clamp MATLAB script

```
Function APD90(filename,APs)
% Written by dkaplan@student.unimelb.edu.au, contact for

load([filename,'.mat']);   % This file contains the matrix 'AveragingPeriod'.
Every row of this matrix contains the voltage trace of a single AP.
% APs is a vector specifying which rows of the matrix AveragingPeriod do not
contain artefacts and can therefore be included in this analysis.

for i=1:numel(APs);
   a=APs(i);
   t=(1:length(AveragingPeriod(a,:)))*2e-5;
   plot(t,AveragingPeriod(a,:))
   % From Threshold
   NTpcentDepolTr=max(AveragingPeriod(a,:))-(max(AveragingPeriod(a,:))-
(Vthresh*1e-3)*0.9); % This calculates the membrane potential at 90% of
threshold.
   [minTr1V iminTr1V]=min(abs(AveragingPeriod(a,1:(numel(t)/2))-
NTpcentDepolTr));                    % Finds the start of the APD90.
   [minTr2V iminTr2V]=min(abs(AveragingPeriod(a,(numel(t)/2):end)-
NTpcentDepolTr));              % Finds the end of the APD90.
hold on
mmm=uint64((numel(t)/2)+iminTr2V);   % Finds the index of the end of the
APD90

   apd90(i)=t(uint64(lll))-t(imin1V);
   apd90Tr(i)=t(uint64(mmm))-t(iminTr1V); % Finds the time difference
difference between the start and the end of the APD90 (the APD90)

   % Plots the traces and marks the start and finish of the APD90
   NTpcentTrVect=zeros(1,length(t));
   NTpcentTrVect(1:end)=NTpcentDepolTr;
   plot(t,NTpcentTrVect,'c--')

plot(t(iminTr1V),AveragingPeriod(a,iminTr1V),'ro',t(uint64(mmm)),AveragingP
eriod(a,uint64(mmm)),'ro')

end
hold off
apd90Tr
apd90Trmean=mean(apd90Tr)
save(['APD90_',filename,'.mat'],'apd90Trmean','apd90Tr')
end
```
