# Supplementary material for: NKX2-5 regulates human cardiomyogenesis via a HEY2 dependent transcriptional network
Source: Nat Commun. 2018 Apr 10;9:1373. doi: 10.1038/s41467-018-03714-x (PMC5893543; doi:10.1038/s41467-018-03714-x)
Supplement: Supplementary file 3 — Description of Additional Supplementary Files(DOCX 15 kb) [file 41467_2018_3714_MOESM3_ESM.docx]

**Description of Additional Supplementary Files**

File Name: Supplementary Data 1

Description: List of deferentially regulated genes between NKX2-5 heterozygotes and NKX2-5 null human embryonic stem cell derived cardiomyocytes.

File Name: Supplementary Data 2

Description: Intersection of NKX2-5 ChIP-seq and NKX2-5-dependant genes to identify putative genes that are direct NKX2-5 regulatory targets.

File Name: Supplementary Data 3

Description: Results of Gene Ontogeny (GO) analysis of genes bound and activated by NKX2-5.

File Name: Supplementary Data 4

Description: Results of Gene Ontogeny (GO) analysis of genes bound and repressed by NKX2-5.

File Name: Supplementary Movie 1

Description: NKX2-5 knockout monolayers display asynchronous contraction across cardiac monolayers. First scene shows representative field of *NKX2-5e^GFP/w^* cells at day 14 of cardiac differentiation. Second scene shows representative field of *NKX2-5^-/-^* cells at day 14 of cardiac differentiation

File Name: Supplementary Movie 2

Description: Example of bioengineered heart muscle. Movement of the supporting posts are used to assess force generation.

File Name: Supplementary Movie 3

Description: Induction of NKX2-5::ER by treatment with the estrogen analogue 4OHT rescues contractility in NKX2-5^-/-^ cardiac monolayers. NKX2-5::ER is expressed by the GAPTrap system. First scene representative field of *NKX2-5^-/-^;GT-NKX2-5::ER* cells, second scene representative field of *NKX2-5^-/-^;GT-NKX2-5::ER* cells cultured in presence of 4OHT both scenes at day 14 of differentiation.
